# Supplementary material for: TerC Proteins Function During Protein Secretion to Metalate Exoenzymes
Source: Res Sq. 2023 May 17:rs.3.rs-2860473. Preprint. [Version 1] doi: 10.21203/rs.3.rs-2860473/v1 (PMC10246235; doi:10.21203/rs.3.rs-2860473/v1)
Supplement: 1 [file NIHPPRS2860473V1-supplement-1.pdf]

EXTENDED DATA

TerC Proteins Function During Protein Secretion to Metalate Exoenzymes

Bixi He[a], Ankita J. Sachla[a], and John D. Helmann*[a]

Table S1. *Bacillus subtilis* stains, primers, and plasmids used in this study.

Figure S1. Comparative growth of WT and FY mutants.

Figure S2. Extracellular protease activities in the supernatants of protease mutants.

Figure S3. Metal ion levels (µM) measured in cell supernatant fractions.

Figure S4. Effect of *meeF*, *meeY* and FY mutations on AprE and AmyQ secretion.

Figure S5. Maximum P_{htrA}-lux promoter activity in stains with or without AmyQ overexpression

Figure S6. MeeF-FLAG and MeeY-FLAG in different mutants.

Figure S7. Comparative phenotypes of mutations affecting protein secretion.

Figure S8. Fold change of transcription level of LTA synthase genes in different strains.

Figure S9. Effects of metal ions of sensitivity to the LtaS inhibitor 1771.

Table S1. Bacillus subtilis stains, primers, and plasmids used in this study.

Strain	Genotype	Construction	Reference
HB27501	*trpC2 attSPβ (WT) CU1065*	Lab strain	Lab stock
HB27502	*CU1065 ΔmeeF*	Lab strain	[1]
HB27503	*trpC2 ΔmeeY*	Lab strain	[1]
HB27504	*trpC2 ΔmeeF ΔmeeY*	Lab strain	[1]
HB27531	*trpC2 aprE::erm*	BGSC-->HB27501	This work
HB27534	*trpC2 nprB::erm*	BGSC-->HB27501	This work
HB27536	*trpC2 nprE::erm*	BGSC-->HB27501	This work
HB27541	*trpC2 mpr::erm*	BGSC-->HB27501	This work
HB27560	*ΔnprE ΔaprE Δepr Δmpr ΔnprB Δvpr Δbpr*		BGSC1A1133
HB27584	*trpC2 ftsH::erm*	BGSC-->HB27501	This work
HB27637	*trpC2 bpr::erm*	BGSC-->HB27501	This work
HB27641	*trpC2 epr::erm*	BGSC-->HB27501	This work
HB27667	*trpC2 ltaS:erm*	BGSC-->HB27501	This work
HB27668	*trpC2 ΔmeeF ltaS:erm*	BGSC-->HB27502	This work
HB27669	*trpC2 ΔmeeY ltaS:erm*	BGSC-->HB27503	This work
HB27670	*trpC2 ΔFY ltaS:erm*	BGSC-->HB27504	This work
HB27680	*trpC2 sacA::*P_{htrA}*-lux-cat*	gDNA HB23650-->HB27501	[2]
HB27681	*trpC2 ΔmeeF sacA::*P_{htrA}*-lux-cat*	gDNA HB23650-->HB27502	This work
HB27682	*trpC2 ΔmeeY sacA::*P_{htrA}*-lux-cat*	gDNA HB23650-->HB27503	This work
HB27683	*trpC2 ΔFY sacA::*P_{htrA}*-lux-cat*	gDNA HB23650-->HB27504	This work
HB27684	*trpC2 amyQ-His::kan*	pKTH10-->HB27501	This work
HB27685	*trpC2 ΔmeeF amyQ-His::kan*	pKTH10 -->HB27502	This work
HB27686	*trpC2 ΔmeeY amyQ-His::kan*	pKTH10 -->HB27503	This work
HB27687	*trpC2 ΔFY amyQ-His::kan*	pKTH10[c]-->HB27504	This work
HB27721	*trpC2 aprE-FLAG::MLS*	*aprE*::pMUTIN-FLAG-->HB27501	This work
HB27722	*trpC2 ΔmeeF aprE-FLAG::MLS*	*aprE*::pMUTIN-FLAG-->HB27502	This work
HB27723	*trpC2 ΔmeeY aprE-FLAG::MLS*	*aprE*::pMUTIN-FLAG-->HB27503	This work
HB27724	*trpC2 ΔFY aprE-FLAG::MLS*	*aprE*::pMUTIN-FLAG-->HB27504	This work
HB27729	*trpC2 sacA::*P_{htrA}*-lux-cat amyQ-His::kan*	pKTH10 -->HB27680	This work
HB27730	*trpC2 ΔmeeF sacA::*P_{htrA}*-lux-cat amyQ-His::kan*	pKTH10 -->HB27681	This work
HB27731	*trpC2 ΔmeeY sacA::*P_{htrA}*-lux-cat amyQ-His::kan*	pKTH10 -->HB27682	This work
HB27732	*trpC2 ΔFY sacA::*P_{htrA}*-lux-cat amyQ-His::kan*	pKTH10 -->HB27683	This work
HB27733	*trpC2 sacA::*P_{sigM}*-lux-cat*	gDNA *sacA::*P_{sigM}*-lux*-->HB27501	[3]

HB27734	*trpC2 ΔmeeF sacA::P_{sigM}-lux-cat*	gDNA *sacA::P_{sigM}-lux*-->HB27502	This work
HB27735	*trpC2 ΔmeeY sacA::P_{sigM}-lux-cat*	gDNA *sacA::P_{sigM}-lux*-->HB27503	This work
HB27736	*trpC2 ΔFY sacA::P_{sigM}-lux-cat*	gDNA *sacA::P_{sigM}-lux*-->HB27504	This work
HB27739	*trpC2 ltaS::erm sacA::P_{sigM}-lux-cat*	gDNA *sacA::P_{sigM}-lux*-->HB27667	This work
HB27740	*trpC2 ltaS::erm ΔmeeF sacA::P_{sigM}-lux-cat*	gDNA *sacA::P_{sigM}-lux*-->HB27668	This work
HB27741	*trpC2 ltaS::erm ΔmeeY sacA::P_{sigM}-lux-cat*	gDNA *sacA::P_{sigM}-lux*-->HB27669	This work
HB27742	*trpC2 ltaS::erm ΔFY sacA::P_{sigM}-lux-cat*	gDNA *sacA::P_{sigM}-lux*-->HB27670	This work
HB27744	*trpC2 ltaSa::spec*	gDNA *ltaSa::spec*-->HB27501	[4]
HB27745	*trpC2 ΔmeeF ltaSa::spec*	gDNA HB27744-->HB27502	This work
HB27746	*trpC2 ΔmeeY ltaSa::spec*	gDNA HB27744-->HB27503	This work
HB27747	*trpC2 ΔFY ltaSa::spec*	gDNA HB27744-->HB27504	This work
HB27750	*trpC2 ltaS::erm ltaSa::spec*	gDNA HB27744-->HB27667	This work
HB27751	*trpC2 ΔmeeF ltaS::erm ltaSa::spec*	gDNA HB27744-->HB27668	This work
HB27752	*trpC2 ΔmeeY ltaS::erm ltaSa::spec*	gDNA HB27744-->HB27669	This work
HB27753	*trpC2 ΔFY ltaS::erm ltaSa::spec*	gDNA HB27744-->HB27670	This work
HB27770	*trpC2 amyE::$P_{spac(hy)}$-meeF-cat*	pPL82-*meeF*-->HB27501	This work
HB27772	*trpC2 amyE::$P_{spac(hy)}$-lmo0991-cat*	pPL82-lmo0991-->HB27501	This work
HB27773	*trpC2 amyE::$P_{spac(hy)}$-lmo0992-cat*	pPL82-lmo0992-->HB27501	This work
HB27774	*trpC2 amyE::$P_{spac(hy)}$-BanTerC-cat*	pPL82-lmoBanTerC-->HB27501	This work
HB27783	*trpC2 ΔFY amyE::$P_{spac(hy)}$-meeF-cat*	pPL82-*meeF*-->HB27504	This work
HB27784	*trpC2 ΔFY amyE::$P_{spac(hy)}$-lmo0991-cat*	pPL82-lmo0991-->HB27504	This work
HB27785	*trpC2 ΔFY amyE::$P_{spac(hy)}$-lmo0992-cat*	pPL82-lmo0992-->HB27504	This work
HB27787	*trpC2 ΔFY amyE::$P_{spac(hy)}$-BanTerC-cat*	pPL82-lmoBanTerC-->HB27504	This work
HB27788	*trpC2 ltaSa::spec sacA::P_{sigM}-lux-cat*	gDNA *sacA::P_{sigM}-lux*-->HB27744	This work
HB27789	*trpC2 ΔmeeF ltaSa::spec sacA::P_{sigM}-lux-cat*	gDNA *sacA::P_{sigM}-lux*-->HB27745	This work
HB27790	*trpC2 ΔmeeY ltaSa::spec sacA::P_{sigM}-lux-cat*	gDNA *sacA::P_{sigM}-lux*-->HB27746	This work
HB27791	*trpC2 ΔFY ltaSa::spec sacA::P_{sigM}-lux-cat*	gDNA *sacA::P_{sigM}-lux*-->HB27747	This work
HB27792	*trpC2 ltaS::erm ltaSa::spec sacA::P_{sigM}-lux-cat*	gDNA *sacA::P_{sigM}-lux*-->HB27750	This work
HB27832	*amyE::$P_{spac(hy)}$-meeY-cat*	pPL82-*meeY*-->HB27501	This work
HB27834	*trpC2 ΔmeeY amyE::$P_{spac(hy)}$-meeY-cat*	pPL82-*meeY*-->HB27503	This work
HB27836	*trpC2 ΔFY amyE::$P_{spac(hy)}$-meeY-cat*	pPL82-*meeY*-->HB27504	This work
HBAS924	*trpC2 meeF-FLAG::MLS*	pMUTIN-FLAG	This work
HBAS943	*trpC2 meeY-FLAG::MLS*	pMUTIN-FLAG	This work
HBAS927	*trpC2 ΔmeeY meeF-FLAG::MLS*	pMUTIN-FLAG	This work

HBAS949	*trpC2 ΔmeeF meeY-FLAG::MLS*	pMUTIN-FLAG	This work
HBAS930	*trpC2 ΔmneP ΔmneS meeF-FLAG::MLS*	pMUTIN-FLAG	This work
HBAS946	*trpC2 ΔmneP ΔmneS meeY-FLAG::MLS*	pMUTIN-FLAG	This work

**Primer name**	**Sequence**	**Purpose**
meeFcheckF	GTGTCATCCATACGGGTGACAA	Deletion check
meeFcheckR	GATTTGAGTGCTTCGCAATCAGCT	
meeYcheckF	GGAGGAAGGCCGTTTCTTGA	Deletion check
meeYcheckR	CGGATGAAACCGTCTTTGCG	
aprEcheckF	CGAGTCTCTACGGAAATAGCGAG	Deletion check
aprEcheckR	CTTGTGAAGATTTTCAGAGGCAGC	
nprBcheckF	GCAGCTCATACCTCCGCTTATAC	Deletion check
nprBcheckR	GATTGGAGCAATCAAACCGTCG	
nprEcheckF	CTCTTGAATGAACCACCACATGAC	Deletion check
nprEcheckR	ATAGCCACGTGACCTGTAGC	
mprcheckF	GCTTGACACTAAAGGAGGGAGATG	Deletion check
mprcheckR	GAAGAATGCGTCTGCGAACTG	
eprcheckF	CACCCGAGTGAATGTGCTCAT	Deletion check
eprcheckR	CATCATTGGGTCTTGCCTGC	
bprcheckF	CAGCGATGTTCTGACAAACCATTC	Deletion check
bprcheckR	TGCCGTGAGCAAAAGCAAAAG	
ftsHcheckF	CTGAGCGCTATCGCAATCTG	Deletion check
ftsHcheckR	TACACGATCAGCGGCTCA	
ltaScheckF	GCGAAACGTTGATTCGACGG	Deletion check
ltaScheckR	GCTGAGGAATTGAGGGCTG	
ftsH700F	ATTGTGACGGACGCAAGCGGTGAAATTATC	PCR insertion
ftsH700R	TCCTGTCGCAATGACCTTTGGTTCCTGT	
aprEFLAGHindIIIF	ATATAAGCTTTCTCACGGCACACATGTAGCCG	Cloning in pMUTIN-FLAG
aprEFLAGKpnIR	ATATGGTACCTTGTGCAGCTGCTTGTACGTTG	
meeFXbalF	CAGTTCTAGAAAAGGAGGAAGGATCATTGGACTTTTTACATCATAT TTTGTCTACG	Cloning in pPL82
meeFBglIIR	CGTTAGATCTTTATTCTTCTTTTGAAGCGGCTGTTT	
meeYXabIF	ATATTCTAGACAAATCACTGCGCTGCGCATATTATTG	Cloning in pPL82
meeYBglIIR	CGTTAGATCTTTACGCCCGTTCACGGGTGCTG	
lmo0991XbalF	CAGTTCTAGAAAAGGAGGAAGGATCAATGGATACAGCAATGATTTT AGAGTAC	Cloning in pPL82
lmo0991BglIIR	CGTTAGATCTTTATTTAGTTGTTTCTGTTTTTTTCTTAC	
lmo0992XbalF	CAGTTCTAGAAAAGGAGGAAGGATCAATGGATGTTTCTATTTGGG GCGAATATG	Cloning in pPL82[5]
lmo0992BglIIR	CGTTAGATCTTCAAACTCTTTTGGTTTTCTTCTC	

BanTerCXbalF	CAGTTCTAGAAAAGGAGGAAGGATCAATGAGTATTTTGCAAGGAATCCTTGATAC	Cloning in pPL82
BanTerCBglIIR	CGTTAGATCTTTATTTATGGTTATTTTTAGTAGCTGCAAC	
ftsHXbalF	CAGTTCTAGAAAAGGAGGAAGGATCAATGAATCGGGTCTTCCGTAATACCATTTTT	Cloning in pPL82
ftsHBglIIR	CGTTAGATCTTTACTCTTTCGTATCGTCTTTCTTTTCTTCTGTT	
meeF-FLAG-HindIII-F	ATATAAGCTTTGGTGGATCAAGGTGCTTGGCGCGCTTTACCTGGCTTG	Cloning in pMUTIN-FLAG
meeF-FLAG-KpnI-R	ATATGGTACCTTCTTCTTTTGAAGCGGCTGTTTGTTCGCGCAC	Cloning in pMUTIN-FLAG
meeY-FLAG-KpnI-F	ATATGGTACCAAGAAGACACACATAAAGAGACGAAGCAAAG	Cloning in pMUTIN-FLAG
meeY-FLAG-KpnI-R	ATATGGTACCCGCCCGTTCACGGGTGCTGTTTTTTTGTTC	Cloning in pMUTIN-FLAG
pMUTIN4-FLAG check-F	ACATCCAGAACAACCTCTGCTAAAATTC	Cloning check; pMUTIN-FLAG
ltaS-F-qPCR	TTCAGTTTTCGTAAACAAAGCGC	qRT-PCR
ltaS-R-qPCR	ATGCCTTCCGCCTTGTACATTTC	qRT-PCR
ltaSa-F-qPCR	CAGCCATTATTATGCTGATTATCG	qRT-PCR
ltaSa-R-qPCR	GCCATGATACTGAAAATCCCGTC	qRT-PCR
yvgJ-F-qPCR	GCCAATTTGGTTCTGACTGTTATC	qRT-PCR
yvgJ-R-qPCR	CTTTTACACTGCTGCCCATATCGCTC	qRT-PCR
yqgS-F-qPCR	ATGCTGATTGCCATTTTATTGATGTG	qRT-PCR
yqgS-R-qPCR	TGCCAGCAATACAAACGTGAC	qRT-PCR

**Plasmid**	**Properties**	**Reference**
pPL82	IPTG-induced overexpression construction	[6]
pKTH10	overexpression of AmyQ	[7]
pMUTIN-FLAG	tagged genes with FLAG sequence at C-terminal	[5]
pBS3C*lux*	luciferase promoter construction	[8]

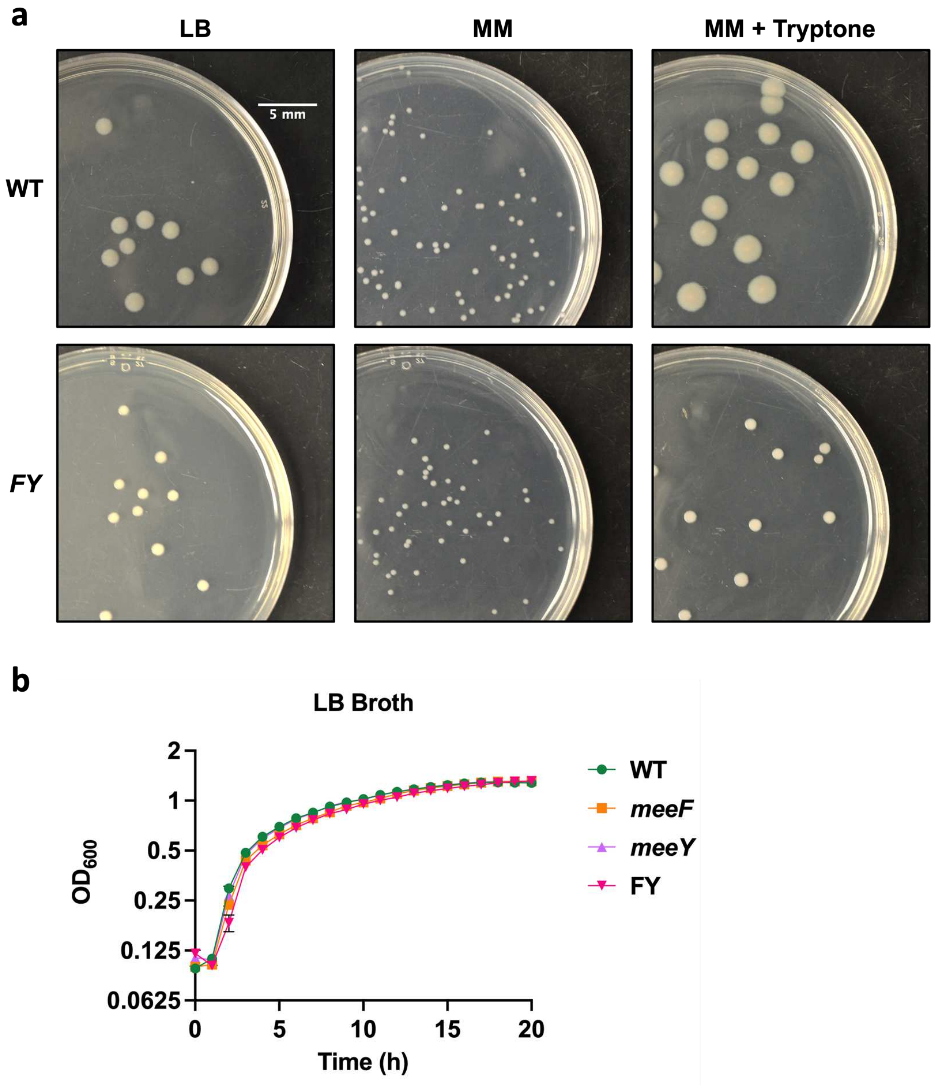

Figure S1. Comparative growth of WT and FY mutants. (**a**) Growth of WT and FY mutants on LB and defined (MM) agar plates. Representative pictures of colony size on different medium plates are shown. Scale bar (5 mm) is indicated and applies across all images. (**b**) Aerobic growth in liquid LB medium. Representative growth curves of different strains (WT, *meeF, meeY, FY*) in liquid LB broth was performed. Cultures were incubated at 37˚C and changes in OD_{600} nm was measured every hour in a 96-well plate reader (Bio-Tek).

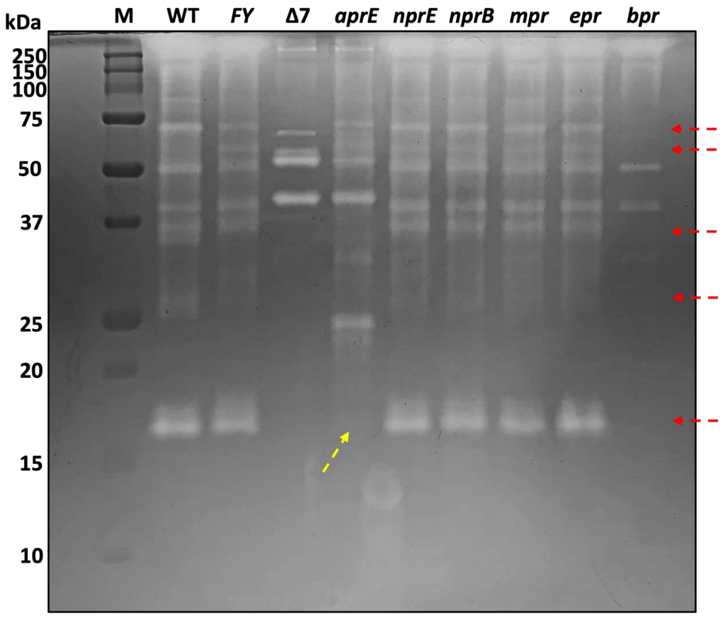

Figure S2. Extracellular protease activities in the supernatants of protease mutants.

Extracellular protease activities in the supernatants were detected by gelatin zymography. Supernatants were collected from overnight cultures with the same cell number. Higher protease activities correspond to clearer bands on the gel matrix. Note that many of the bands lacking in the *bpr* null mutant were previously proposed to be processed products of the large Bpr protease (red arrows)[9]. The 17 kDa processed Bpr product is also missing in the *aprE* mutant (yellow arrow), suggesting that the AprE protease is involved in Bpr processing, as suggested[9]. The presence of the 17 kDa band in the FY mutant suggests that both Bpr and AprE are still secreted in this strain, although overall protease activity is reduced.

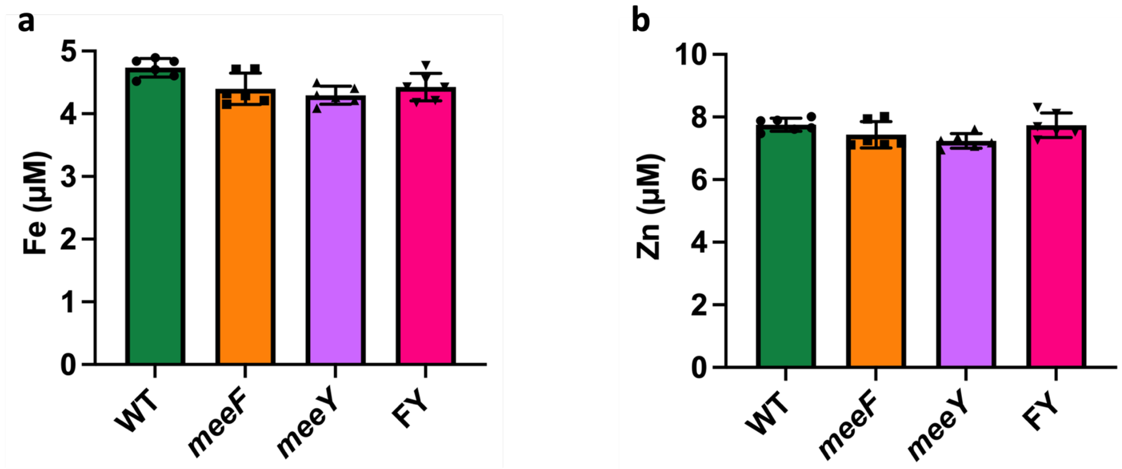

C. Table. Metal levels (µM) in LB or spent supernatants detected by ICP-MS

	LB broth	WT	*meeF*	*meeY*	*FY*
**Mn**	0.126±0.009	0.022±0.002	0.017±0.005	0.021±0.003	0.0032±0.0009
**Fe**	5.05±0.62	4.73±0.15	4.40±0.25	4.30±0.14	4.43±0.22
**Zn**	8.20±0.87	7.75±0.21	7.43±0.42	7.24±0.23	7.74±0.39

Figure S3. Metal ion levels (µM) measured in cell supernatant fractions. Overnight grown cells were used to collect supernatants and Fe (**a**) and Zn (**b**) levels were detected using ICP-MS analysis. Data is from three independent experiments and presented as mean ± SD. In contrast with Mn levels (Fig. 2b), there was little change in residual Fe and Zn in the supernatant fractions. (**c**) Summary Table: Samples were collected and analyzed as in Fig. 2b, S3a and S3b. Samples were from three independent experiments and data is presented as mean ± SD.

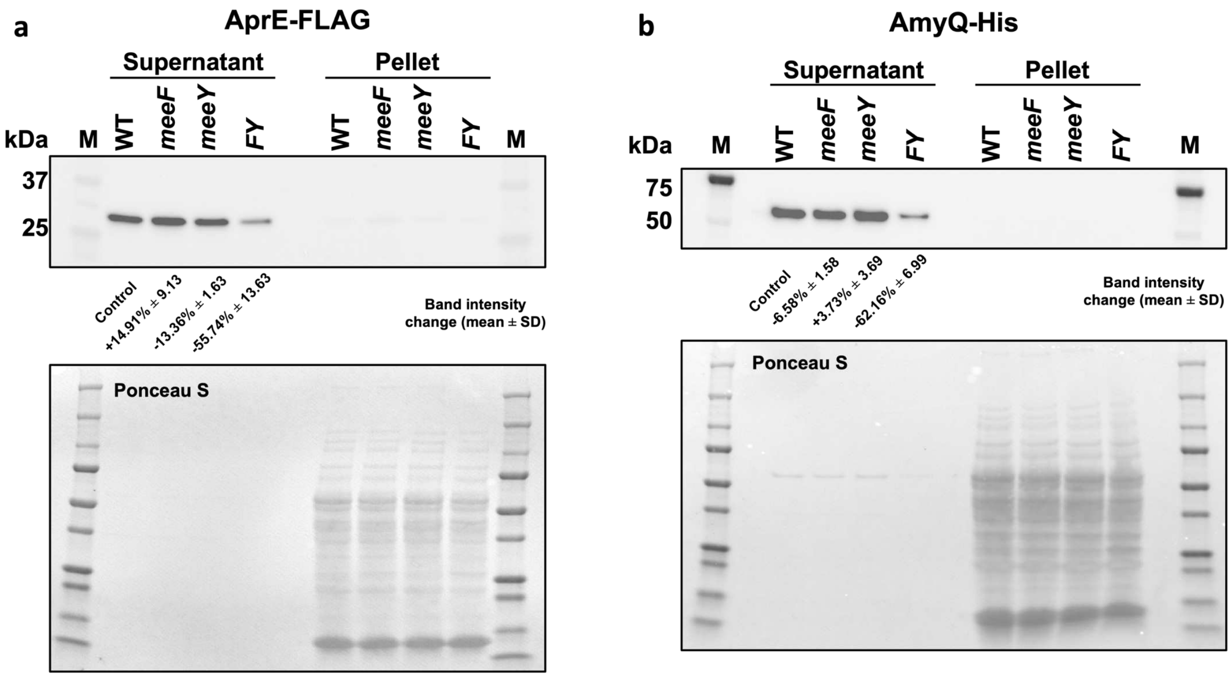

Figure S4. Effect of *meeF*, *meeY* and FY mutations on AprE and AmyQ secretion. (**a**) The level of AprE-FLAG was measured using immunoblotting. (**b**) Defective secretion of heterologous (AmyQ-His) protein is depicted for FY. Sample collection was described in Fig 3. The protein membranes were stained by Ponceau after protein transfer. Ponceau-stained images as the loading control were taken using GelDoc gel imaging system. Band intensities were measured from three independent experiments. Band intensity change was calculated as "change = (sample - control) / control * 100%".

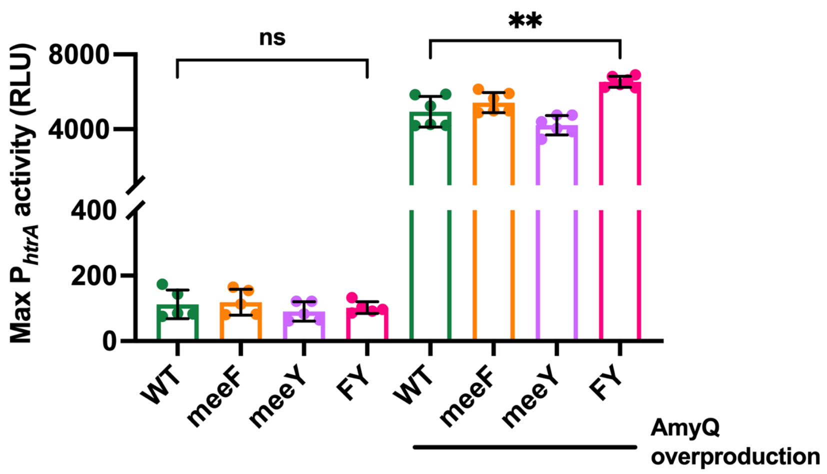

Figure S5. Maximum P$_{htrA}$-lux promoter activity in stains with or without AmyQ overexpression. Expression of the CssRS secretion stress response was monitored using an P$_{htrA\text{-}lux}$ transcriptional reporter. Cells were grown in LB medium at 37˚C. AmyQ was overexpressed in the strains from pKTH10[7]. Data are presented as mean ± SD. ns, no significant difference, P=0.6540; **, P=0.0035, P value was calculated using Welch's t test, two-tailed.

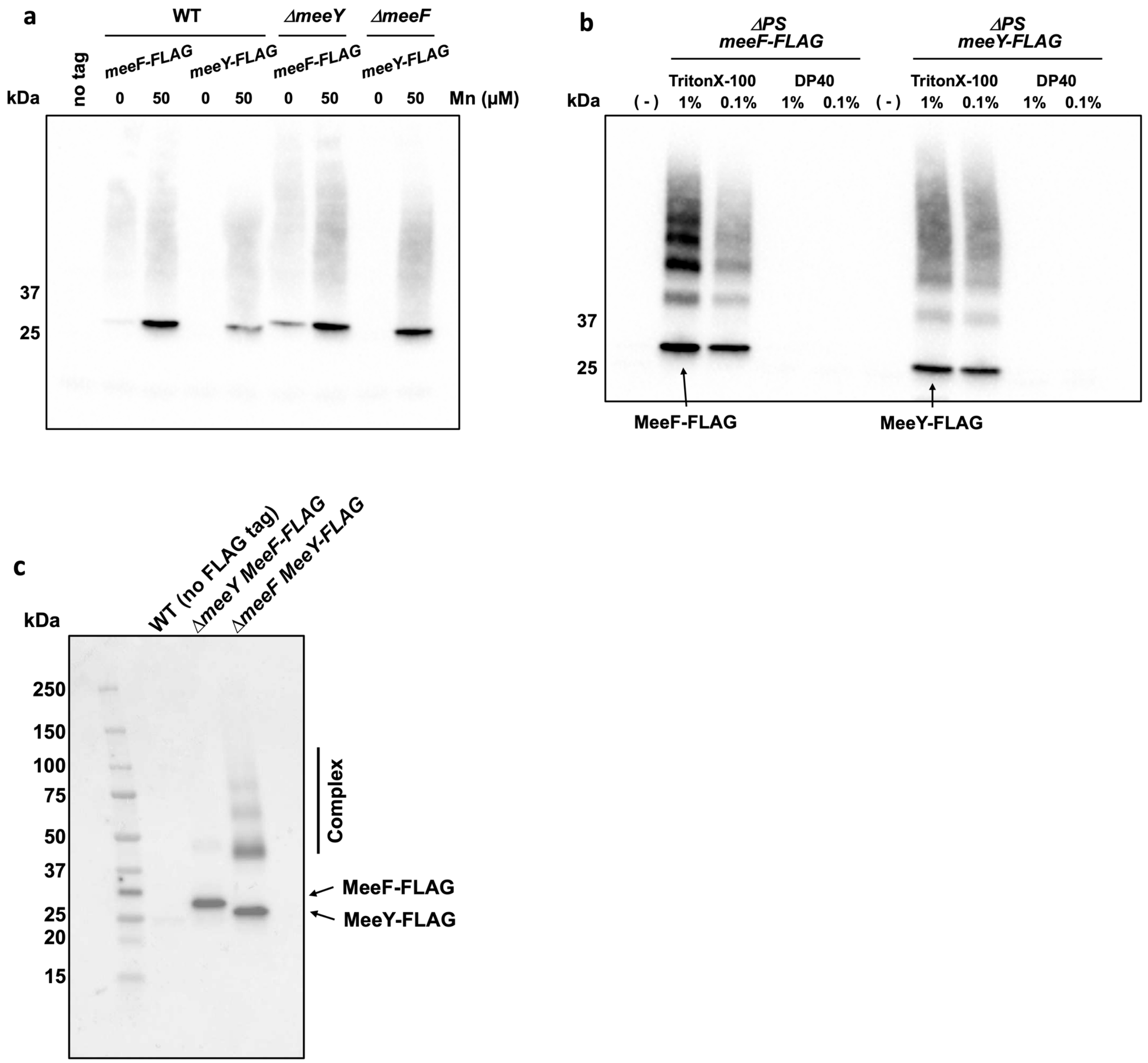

Figure S6. MeeF-FLAG and MeeY-FLAG in different mutants. (**a**) MeeF-FLAG or MeeY-FLAG levels in different stain backgrounds as measured using immunoblotting. Cells were collected from overnight cultures with or without 50 μM Mn. (**b**) Representative immunoblot analysis of MeeF-FLAG and MeeY-FLAG co-IP samples in an efflux deficient *mneP mneS* (Δ*PS*) background. Different detergents were used for cell lysis (1% or 0.1% Triton X-100, and 1% or 0.1% DP40). Proteins were eluted from magnetic beads by heating at 95˚C for 10 min. (-) WT is the control without FLAG tag. (**c**) Co-Immunoprecipitation of MeeF and MeeY interact.

Representative Western-blot analysis of MeeF / MeeY - FLAG (bait) is shown following pull down of cell lysates prepared with 1% Triton X-100 using anti-FLAG-magnetic beads and subsequent elution. The WT strain without FLAG tag was used as a control to diminish false positive protein interactions. Proteins (prey) that identified in the immunoprecipitate (and not present with the control, untagged strain) are considered interacting protein partners of MeeF/MeeY-FLAG. The detection of lower mobility bands represents a possible complexes stable during electrophoresis.

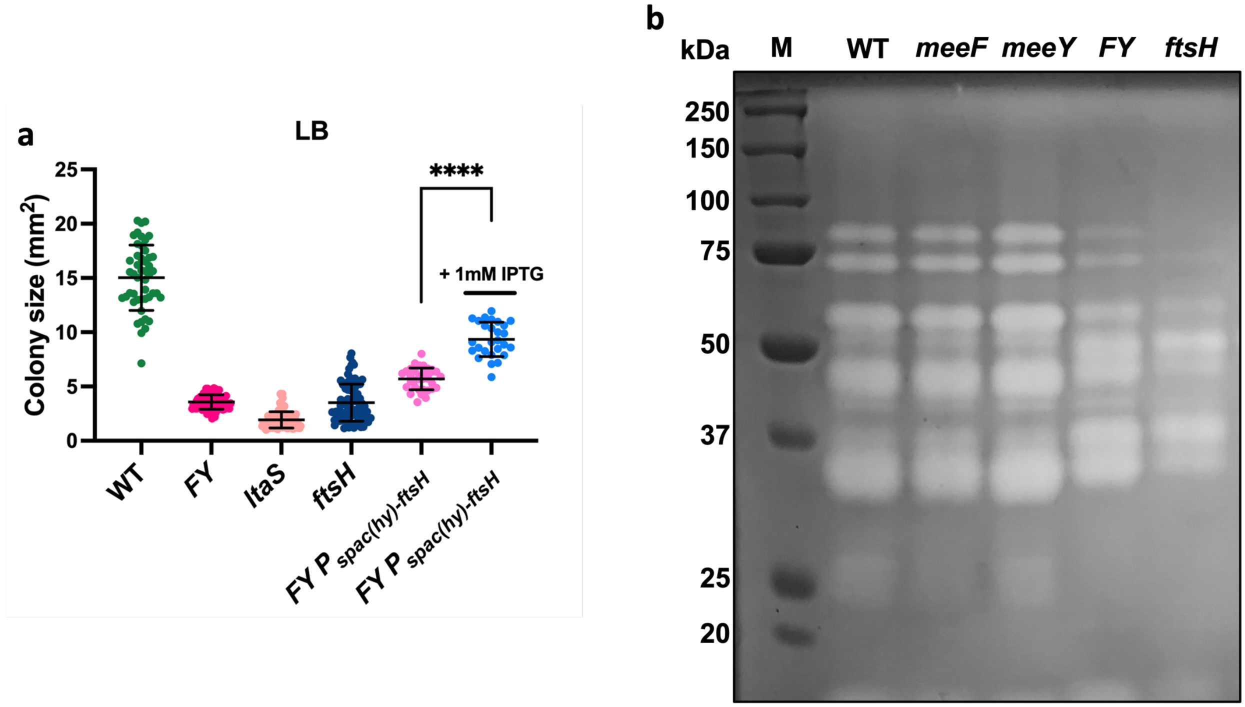

Figure S7. Comparative phenotypes of mutations affecting protein secretion. (**a**) Colony size (mean ± SD) of the indicated strains on LB medium. At least 25 isolated colonies from two independent experiments were measured for each strain. Growth of the FY mutant strain was significantly improved upon addition of 1 mM IPTG (**** = P<0.0001). P value was calculated using Welch's t test, two-tailed. (**b**) Protease secretion monitored by zymography. Supernatants were collected from overnight cultures with equal cell density. Higher protease activities represent clearer bands on gel matrix. *ftsH* mutant showed defective protease activities but different pattern compared to FY. M, marker.

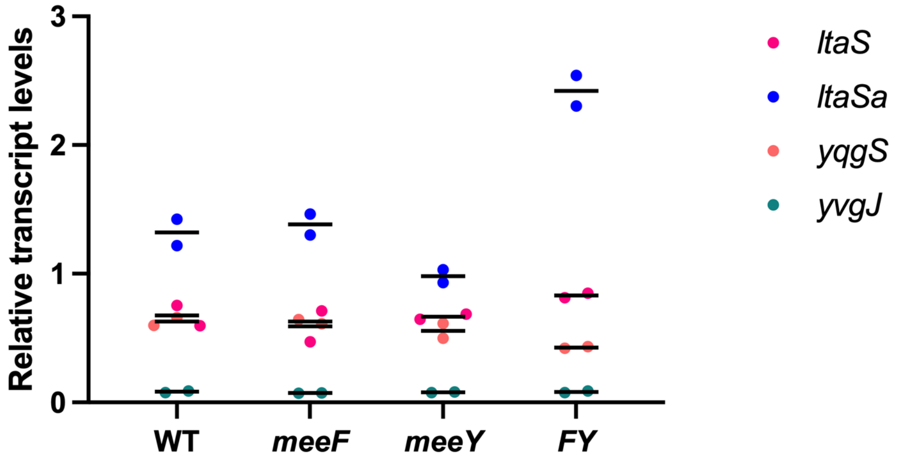

Figure S8. Fold change of transcription level of LTA synthase genes in different strains. Transcription levels (mean ± SD) of four LTA synthases or primase *ltaS*, *ltaSa*, *yqgS* and *yvgJ* in different stains (WT, *meeF*, *meeY* and FY). Samples are from two independent experiments. FY mutants showed similar *ltaS* expression and higher *ltaSa* transcription levels compared to WT, which is consistent with the hypothesis that FY mutant has normal expression of *ltaS* but decreased LtaS activity.

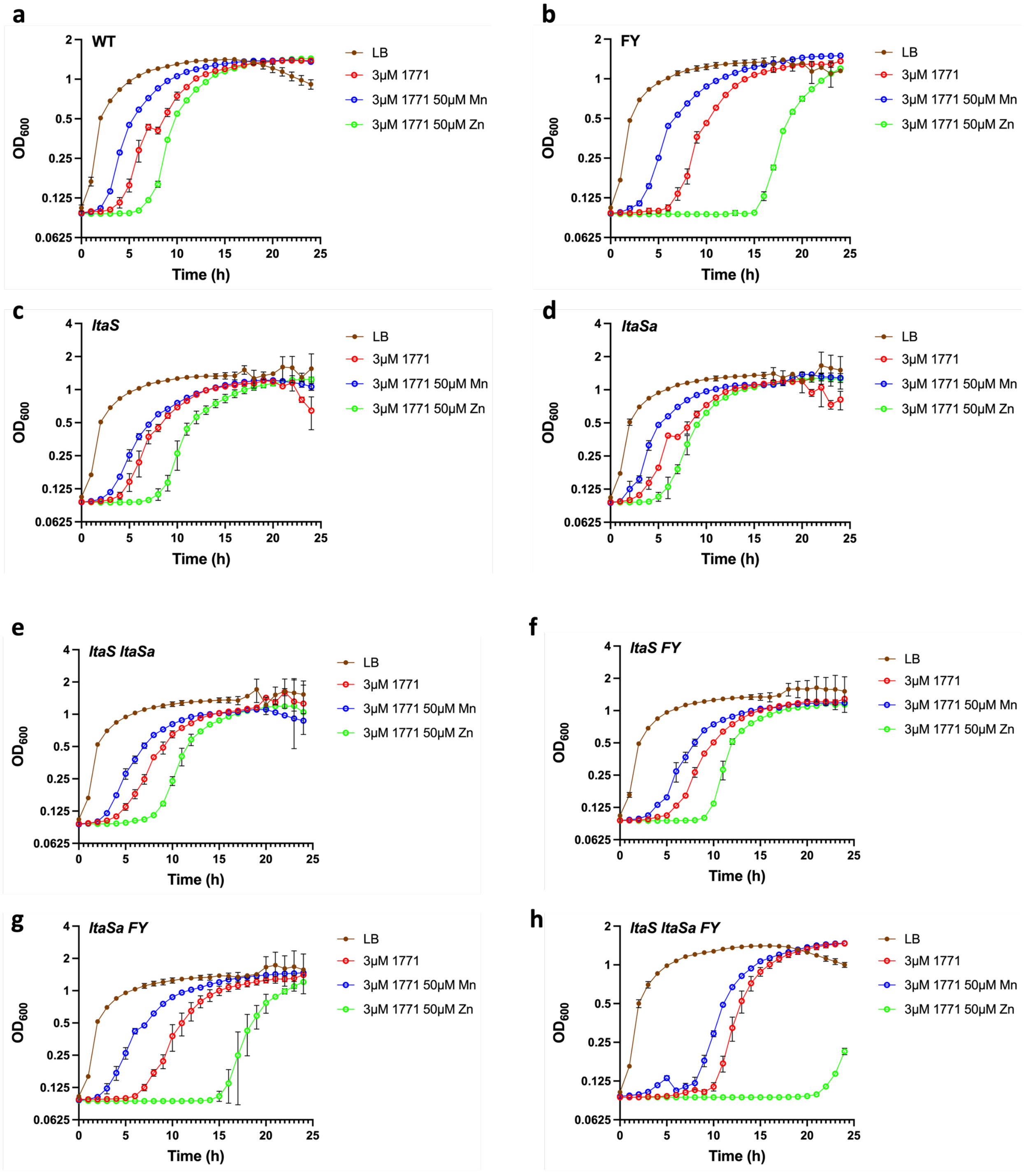

Figure S9. Effects of metal ions of sensitivity to the LtaS inhibitor 1771.

(**a**)-(**h**), Aerobic growth of different strains in LB broth with or without 3 µM 1771, 50 µM Mn or 50 µM Zn. Data is representative of three independent cultures and presented as mean ± SD.

REFERENCES

1. Paruthiyil, S., Pinochet-Barros, A., Huang, X. & Helmann, J.D. Bacillus subtilis TerC Family Proteins Help Prevent Manganese Intoxication. *J Bacteriol* **202**(2020).
2. Zhao, H., Sachla, A.J. & Helmann, J.D. Mutations of the Bacillus subtilis YidC1 (SpoIIIJ) insertase alleviate stress associated with sigmaM-dependent membrane protein overproduction. *PLoS Genet* **15**, e1008263 (2019).
3. Zhao, H., Roistacher, D.M. & Helmann, J.D. Aspartate deficiency limits peptidoglycan synthesis and sensitizes cells to antibiotics targeting cell wall synthesis in Bacillus subtilis. *Mol Microbiol* **109**, 826-844 (2018).
4. Sachla, A.J. & Helmann, J.D. A bacterial checkpoint protein for ribosome assembly moonlights as an essential metabolite-proofreading enzyme. *Nat Commun* **10**, 1526 (2019).
5. Kaltwasser, M., Wiegert, T. & Schumann, W. Construction and Application of Epitope- and Green Fluorescent Protein-Tagging Integration Vectors for Bacillus subtilis. *Applied and Environmental Microbiology* **68**, 2624-2628 (2002).
6. Quisel, J.D., Burkholder, W.F. & Grossman, A.D. In vivo effects of sporulation kinases on mutant Spo0A proteins in Bacillus subtilis. *Journal of bacteriology* **183**, 6573-6578 (2001).
7. Palva, I. Molecular cloning of alpha-amylase gene from Bacillus amyloliquefaciens and its expression in B. subtilis. *Gene* **19**, 81-7 (1982).
8. Radeck, J. et al. The Bacillus BioBrick Box: generation and evaluation of essential genetic building blocks for standardized work with Bacillus subtilis. *J Biol Eng* **7**, 29 (2013).
9. Kodama, T., Endo, K., Ara, K., Ozaki, K. & Sekiguchi, J. Zymography of extracellular proteases in Bacillus subtilis. *Int. J. Biosci. Biotechnol.* **1**, 60-66 (2013).
